# Histamine modulation of urinary bladder urothelium, lamina propria and detrusor contractile activity via H1 and H2 receptors

**DOI:** 10.1038/s41598-019-40384-1

**Published:** 2019-03-07

**Authors:** Zane Stromberga, Russ Chess-Williams, Christian Moro

**Affiliations:** Centre for Urology Research, Faculty of Health Sciences and Medicine, Bond University, Queensland, 4229 Australia

## Abstract

The mechanisms underlying bladder contractile disorders such as overactive bladder are not fully understood, and there is limited understanding of the receptor systems modulating spontaneous bladder contractions. We investigated the potential for histamine to have a role in mediating contractility of the urothelium with lamina propria (U&LP) or detrusor via the H1-H4 histamine receptor subtypes. Isolated strips of porcine U&LP or detrusor smooth muscle were mounted in gassed Krebs-bicarbonate solution and responses to histamine obtained in the absence and presence of selective receptor antagonists. The presence of histamine increases the frequency of U&LP spontaneous phasic contractions and baseline tensions. In response to histamine, H1-antagonists pyrilamine, fexofenadine and cyproheptadine were effective at inhibiting contractile responses. Cimetidine (H2-antagonist) enhanced increases in baseline tension in response histamine, whereas amthamine (H2-agonist) induced relaxation. Although thioperamide (H3/H4-antagonist) increased baseline tension responses to histamine, selective H1/H2-receptor antagonism revealed no influence of these receptors. In detrusor preparations, pyrilamine, fexofenadine and cyproheptadine were effective at inhibiting baseline tension increases in response to histamine. Our findings provide evidence that histamine produces contractile responses both in the U&LP and detrusor via the H1-receptor, and this response is significantly inhibited by activation of the H2-receptor in the U&LP but not the detrusor.

## Introduction

The underlying mechanisms of bladder contractile disorders such as overactive bladder (OAB) are unclear. However, it is known that detrusor smooth muscle provides the force for contractions during voiding. In recent years, interest has been building on the role of the urothelium and lamina propria (U&LP) in regulating and modulating overall bladder contractile activity. Among other functions, U&LP is capable of releasing various mediators when activated by chemical or mechanical stimuli such as acetylcholine which can influence detrusor contractions^1^. It is also capable of responding to external stimuli such as noradrenaline^2^ or nitric oxide^3^ and can release agents such as ATP, which may contribute to overactive bladder^4^. Research also suggests that U&LP is capable of developing spontaneous phasic contractile activity in the absence of any external stimulation, and this activity can potentially influence the bladder as a whole^1^.

Bladder contractions are mainly controlled by the parasympathetic nerves^5^ which release acetylcholine to activate M3 muscarinic receptors on the detrusor smooth muscle^6^. Antimuscarinic treatment has been used as the first-line pharmacology intervention for many years, however, there is a need for alternative treatment options due to the large number of side-effects associated with antimuscarinic use^7^ and the low rates of patient adherence^8^. One alternative target involved in the pathogenesis of contractile dysfunctions, such as OAB may be the inflammatory mediators released from mast cells at sites of inflammation.

Mast cells play an important role in immediate allergic reactions and during inflammation^9^. Upon activation, they release and synthesise potent inflammatory mediators including histamine, prostaglandins, proteases, cytokines that act on smooth muscle, connective tissue, mucous glands and other inflammatory cells^10^. In the urinary bladder, mast cells can be found in the urothelium, lamina propria and smooth muscle layers of the bladder wall^11–13^ and have previously been associated with the pathogenesis of interstitial cystitis^11,14^ and OAB^15^. Furthermore, upregulation of chemoattractant protein-1 (MCP-1) which causes degranulation of mast cells has been observed in patients with OAB^16^. A histological investigation of 179 biopsies obtained from patients with OAB found signs of chronic inflammation in 70% of biopsies with inflammatory cells having infiltrated the lamina propria in 98% of biopsies and urothelium in 18%^17^. Therefore, the involvement of histamine is of great interest as it is the predominant mediator involved in the mechanisms of acute allergy and inflammation.

Histamine exerts its function by binding to four different G protein-coupled receptors, namely H1, H2, H3 and H4^18^. Both H1 and H2 receptors are co-expressed in most tissues and cell types, including smooth muscle, epithelial tissue, neurons and various white blood cells^19^. Western blot analysis of cultured human detrusor cells has revealed the presence of all four histamine receptors subtypes: H1, H2, H3 and H4^20^, although functional roles of these receptor subtypes are still not fully understood. There is also evidence that histamine is involved in the modulation of bladder contractile activity via the activation of H1 receptors in guinea pig^21–23^ and rabbit detrusor muscle^24^. It has also been suggested that responses to histamine may stimulate acetylcholine release from sites proximal to the muscle^25^, or influence purinergic neurotransmission on nearby nerves^26^. This study aims to investigate the effect of histamine on the contractility of U&LP and detrusor smooth muscle and identify the histamine receptor subtypes responsible for mediating contractile responses.

## Materials and Methods

### Tissue preparation

The urinary bladders from Large White-Landrace pigs were acquired from the local commercial abattoir after slaughter for the routine commercial provision of food. All methods were carried out in accordance with relevant Australian guidelines and regulations, and all experimental protocols were in accordance the Australian Code of Practice for the Care and Use of Animals for Scientific Purposes. Urinary bladder was separated from the urethra and the ureters removed. Isolated strips (10 mm × 5 mm) were removed from the anterior wall of the bladder dome region and urothelium with lamina propria dissected from the underlying detrusor layer, consistent with past studies^1^. Adjacent pieces of U&LP and detrusor were suspended in pairs in 10 mL organ baths (Labglass, Brisbane, Australia) containing Krebs-bicarbonate solution (NaCl 118.4 mM, NaHCO_3_ 24.9 mM, CaCl_2_ 1.9 mM, MgSO_4_ 2.41 mM, KCl 4.6 mM, KH_2_PO_4_ 1.18 mM and D-glucose 11.7 mM) at 37 °C and perfused with a gas mixture of 95% oxygen and 5% carbon dioxide. Tissue strips were washed three times and tension readjusted to approximately 2 g, where this was maintained as the baseline tension. A single dose of agonist was added to the tissue both in the presence and absence of antagonists, and concentrations selective for each receptor subtype determined using affinity values appearing in the literature. Tissue strips that were exposed to antagonists and tissues treated with vehicle control were incubated for 30 minutes to allow full equilibration with the receptor. Baseline tension and the frequency and amplitude of spontaneous phasic contractions were recorded simultaneously using isometric force transducers (MCT050/D, ADInstruments, Castle Hill, Australia) on a Powerlab system using Labchart v7 software (ADInstruments). Changes in tension were measured in grams, frequency was expressed as the number of spontaneous phasic contractions per minute (cycles/min) and amplitude was expressed in grams (lowest point of spontaneous phasic contraction to peak). Data was graphed and analysed using GraphPad Prism version 7.00 for Windows (GraphPad Software, La Jolla California USA) and results shown as the mean change ± SEM. Responses between control and experimental tissues were compared using Student’s *t*-test with p < 0.05 considered significant.

### Histamine agonist effects on baseline tension, amplitude and spontaneous phasic contractions

A single dose of histamine (100 µM, Sigma Aldrich, Missouri, USA) or amthamine (100 µM, H2 agonist, Sigma Aldrich, Missouri, USA) was applied to the tissue after a 30-minute equilibration period. Baseline tension, amplitude and spontaneous phasic contractions of U&LP were measured before the application of the agonist and 2 minutes after. The baseline tension for detrusor was measured at the same time-points as used for U&LP.

### The effects of histamine on baseline tension, amplitude and spontaneous phasic contractions in the presence of histamine receptor antagonists

The H1 receptor antagonists pyrilamine (30 nM, Cayman Chemicals, Michigan, USA), fexofenadine (1 µM, Cayman Chemicals, Michigan, USA) and cyproheptadine (30 nM, Cayman Chemicals, Michigan, USA), the H2 receptor antagonist cimetidine (1 µM, Sigma Aldrich, Missouri, USA) and the dual H3&H4 receptor antagonist thioperamide (1 µM, Sigma Aldrich, Missouri, USA) were separately applied to tissues. After 30-minute incubation with antagonists, a single dose of histamine (100 µM) was applied to the tissues. In another group of experiments, a combination of antagonists was used. Histamine responses to pyrilamine (30 nM) treated tissues were compared with a combination of pyrilamine (30 nM) and cimetidine (1 µM) treated tissues; cimetidine (1 µM) treated tissues were compared with a combination of pyrilamine (30 nM) and cimetidine (1 µM) treated tissues; combination of pyrilamine (30 nM) and cimetidine (1 µM) treated tissues were compared with a combination of pyrilamine (30 nM), cimetidine (1 µM) and thioperamide (1 µM) treated tissues.

## Results

### Influence of histamine receptor agonists in U&LP and detrusor

In the absence of histamine or any antagonists, strips of urothelium/lamina propria (U&LP) developed spontaneous phasic contractions at a frequency of 3.56 ± 0.13 cycles per min^−1^ (cpm) with an amplitude of 0.46 ± 0.03 g (n = 48). When histamine (100 µM) was added to the tissues, U&LP baseline tension increased by 0.98 ± 0.13 g (p < 0.001, n = 48). Additionally, frequency of spontaneous phasic contractions increased by 1.32 ± 0.25 cpm (p < 0.001, n = 48) and amplitude decreased by 22.9 ± 5.8% (p < 0.001, n = 48) during the peak response to histamine (100 µM). Baseline tension, frequency and amplitude of spontaneous phasic contractions in response to histamine was not affected by the presence of the muscarinic receptor antagonist atropine (1 µM, Sigma Aldrich, Missouri, USA).

When H2 agonist amthamine (1 μM) was added to strips of U&LP that were left to equilibrate at a tension of approximately 2 g in the absence of any agonists and antagonists, resting baseline tension decreased by 0.13 ± 0.02 g (p < 0.01, n = 12) 5 minutes after the addition of agonist and by 0.22 ± 0.04 g after 20 minutes (p < 0.01, n = 12).

In detrusor preparations, an increase in baseline tension of 0.99 ± 0.27 g (p < 0.001, n = 48) was observed in response to histamine (100 µM). The addition of H2 agonist amthamine (1 µM) had no influence on the baseline tension in detrusor preparations.

### Influence of selective histamine receptor antagonists in U&LP

**Figure 1:**
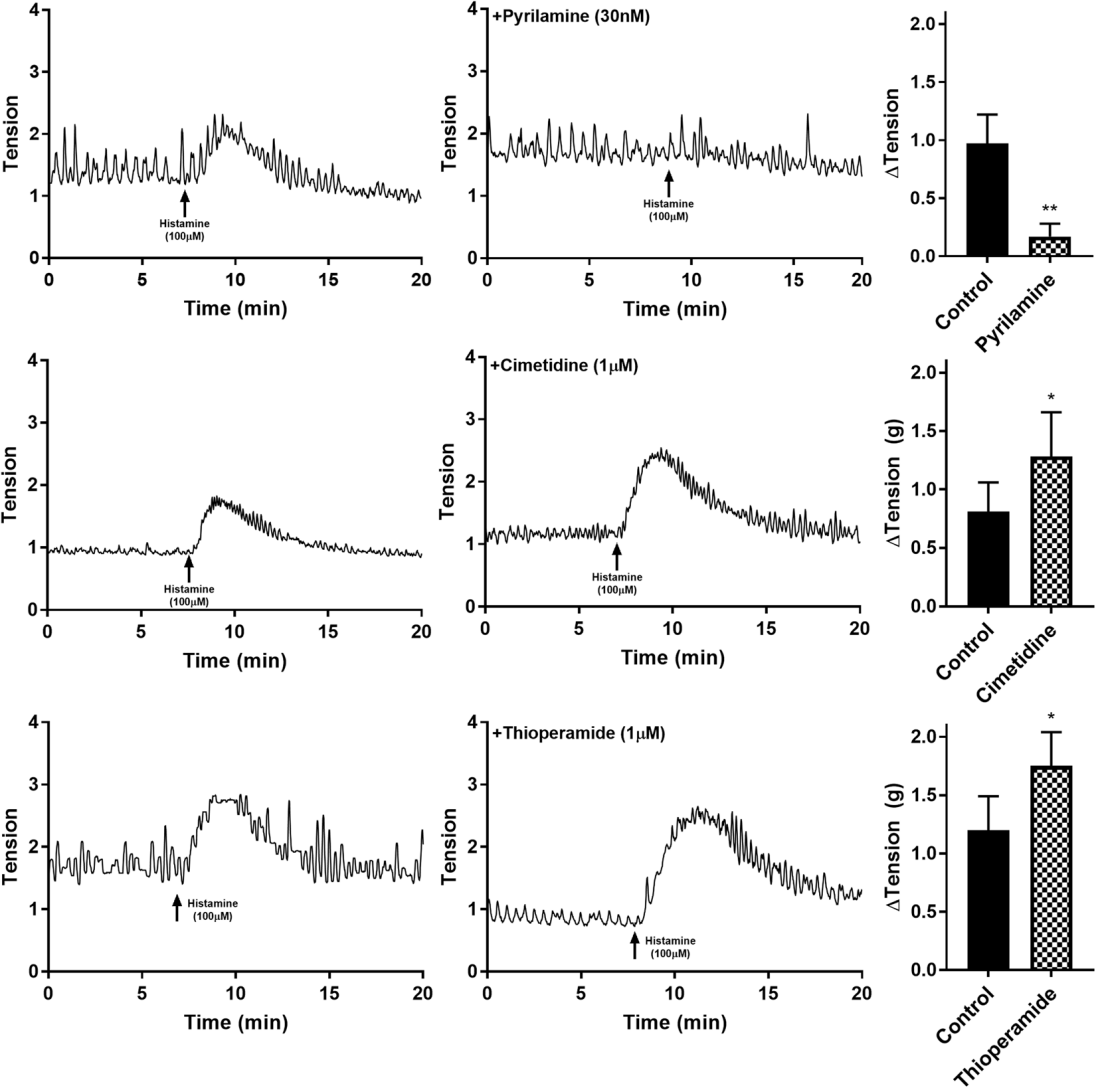
U&LP responses to histamine (100 µM) as control (left) and in the presence of histamine receptor antagonists pyrilamine, cimetidine and thioperamide (right).

The responses to histamine were observed in the presence of three selective histamine receptor antagonists: pyrilamine, cimetidine and thioperamide. In the presence of pyrilamine (H1 antagonist, 30 nM), increases in the baseline tension and frequency of spontaneous phasic contractions in response to histamine (100 µM) were significantly inhibited (n = 8, p < 0.05 for both). Histamine (100 µM) in the presence of pyrilamine caused an increase in baseline tension by 0.17 ± 0.11 g (Fig. 1) and in the frequency of spontaneous phasic contractions by 0.15 ± 0.25 cpm (Table 1). Furthermore, when the alternative H1-selective antagonists fexofenadine (1 µM, n = 8) and cyproheptadine (30 nM, n = 8) were added to the U&LP tissue strips, inhibitions to baseline tension and frequency of spontaneous phasic contraction increases (Table 1) in response to histamine (100 µM) were also observed (p < 0.01 for both).

**Table 1:** U&LP responses to histamine (100 µM) in the presence of histamine receptor antagonists (mean change ± SEM).

Antagonist	Conc.	ΔTension (g)		ΔFrequency (cpm)		n
		Absence	Presence	Absence	Presence	
Pyrilamine	30 nM	0.97 ± 0.25	0.17 ± 0.11^*^	1.67 ± 0.26	0.24 ± 0.22**	8
Cimetidine	1 μM	0.81 ± 0.25	1.28 ± 0.38^*^	1.20 ± 0.65	1.27 ± 1.08	8
Thioperamide	1 μM	1.20 ± 0.29	1.75 ± 0.29^*^	1.49 ± 0.63	1.76 ± 0.55	6
Cyproheptadine	30 nM	1.19 ± 0.38	0.42 ± 0.16^*^	1.87 ± 0.78	0.56 ± 0.28^*^	8
Fexofenadine	1 μM	0.89 ± 0.24	−0.03 ± 0.02**	1.11 ± 0.28	−0.09 ± 0.14^*^	8

*p < 0.05, **p < 0.01. Paired Student’s *t*-test.

Cimetidine (H2 antagonist, 1 µM) caused a significant increase of 0.81 ± 0.25 g above the baseline tension in response to histamine (100 µM) (n = 8, p < 0.05, Fig. 1) and an increase in the frequency of spontaneous phasic contractions of 1.41 ± 0.94 cpm (Table 1). The addition of thioperamide (H3/H4 antagonist, 1 µM) resulted in the tissue achieving significantly greater contractions to histamine (100 µM, n = 6) than in control samples, resulting in a 1.75 ± 0.29 g increase in tension from baseline tension (p < 0.05), with no influence on the frequency of spontaneous contractions (Table 1). None of the antagonists had any influence on the amplitude of phasic spontaneous contractions in response to histamine (100 µM).

**Figure 2:**
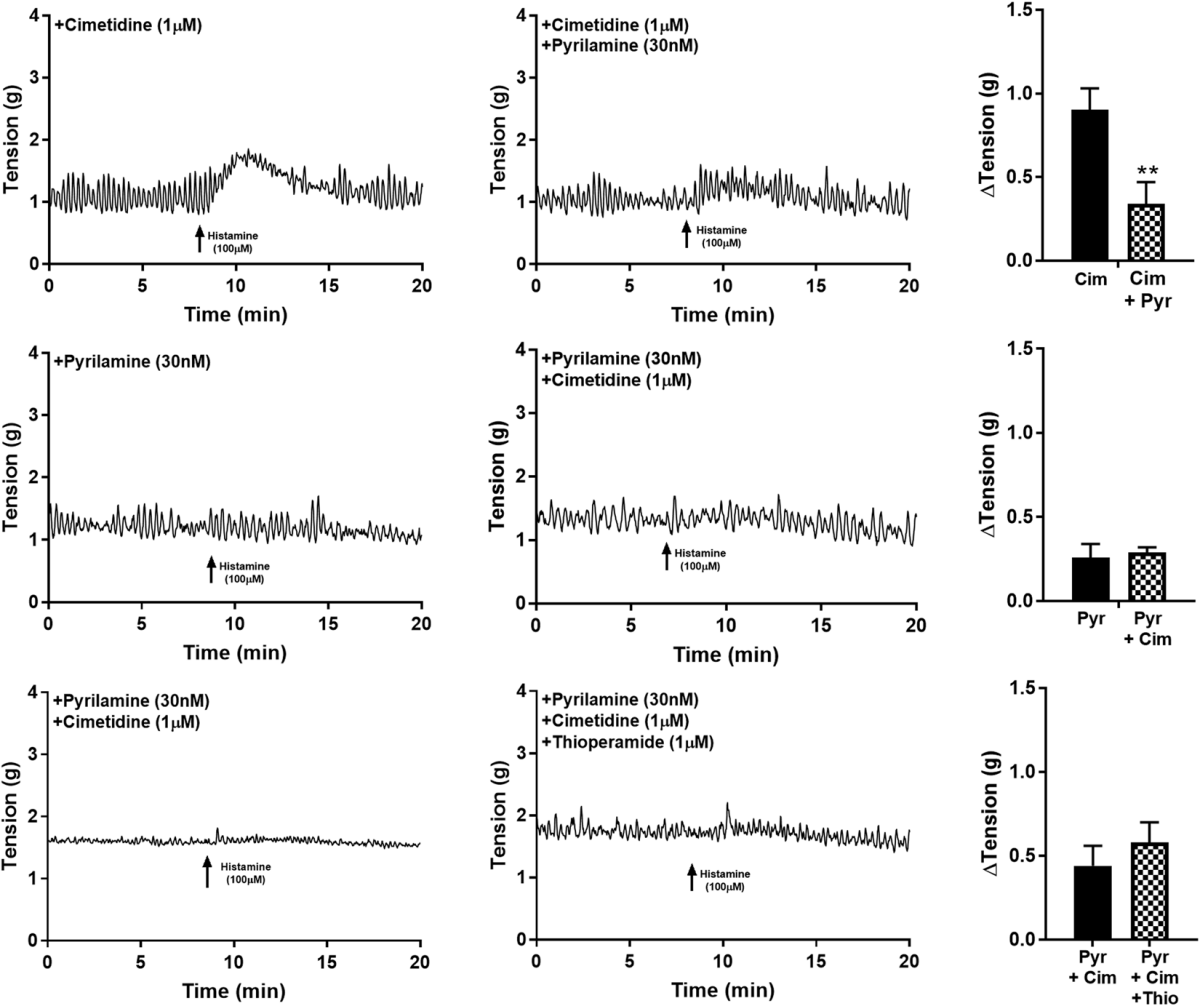
U&LP responses to histamine (100 µM) in the presence of more than one histamine receptor antagonist. **p < 0.001.

In experiments where combinations of antagonists were used, the ability of cimetidine (1 µM) to enhance contraction to histamine (100 µM) was abolished when tissues were also treated with pyrilamine (30 nM, n = 8, p < 0.001, Fig. 2). Alternatively, the effectiveness of pyrilamine (30 nM) inhibiting histamine responses (100 µM) was equally effective in baths containing a combination of pyrilamine (30 nM) and cimetidine (1 µM, n = 8, Fig. 2).

To isolate the potential influence of H3 and H4 receptors, thioperamide (1 µM) was added to tissues pre-treated with pyrilamine (30 nM) and cimetidine (1 µM, n = 8). Responses to histamine (100 µM) in the presence of thioperamide (1 µM) exhibited a small but observable increase in baseline tension, however, this increase was not significant.

### Influence of selective histamine receptor antagonists on the detrusor

**Figure 3:**
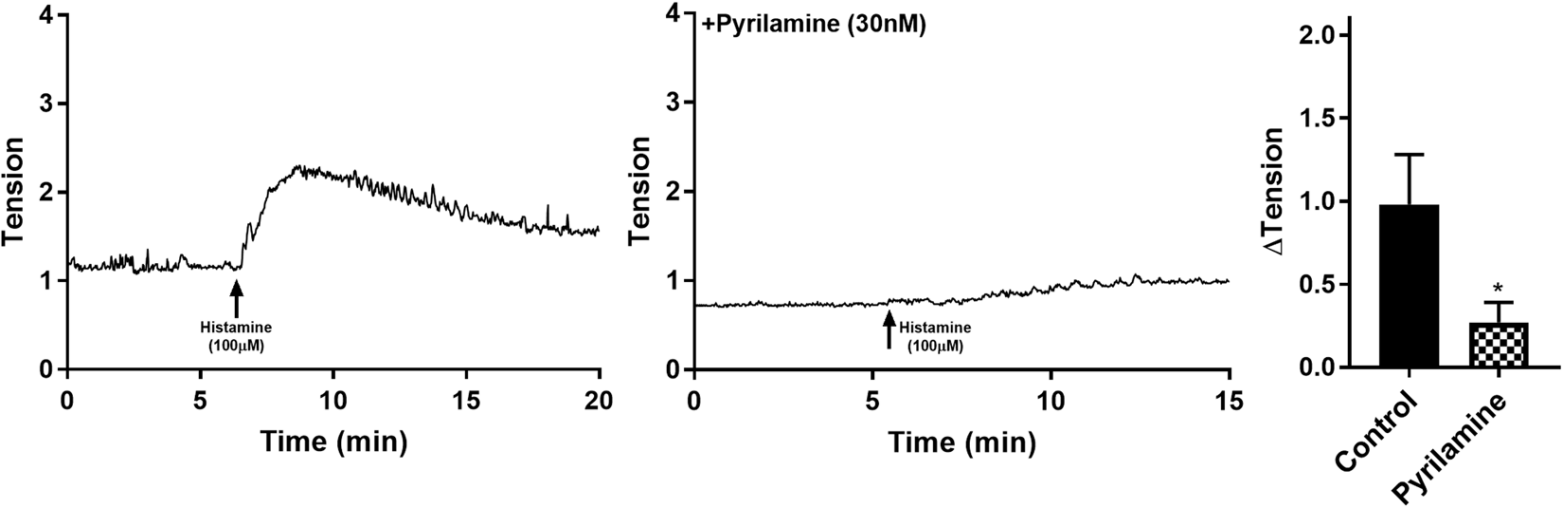
Detrusor responses to histamine (100 µM) as a control (left) and in the presence of pyrilamine (right, 30 nM).

In the presence of 30 nM pyrilamine (H1 antagonist), responses to histamine (100 µM) were significantly inhibited in comparison to the control tissues (n = 8, p < 0.05, Table 2, Fig. 3). In the presence of 1 µM cimetidine (H2 antagonist, n = 8) and 1 µM thioperamide (H3/H4 blocker, n = 4) no significant differences between the control and experimental tissues were observed (Table 2, Fig. 3). The addition of the alternative H1-selective antagonist fexofenadine (1 µM, n = 8) and cyproheptadine (30 nM, n = 8) showed inhibited contractile responses to histamine (p < 0.005, Table 2, Fig. 3).

**Table 2:** Detrusor responses to histamine (100 µM) in the presence of histamine receptor antagonists (mean change ± SEM).

Antagonist	Conc.	ΔTension (g)		n
		Absence	Presence	
Pyrilamine	30 nM	0.98 ± 0.30	0.27 ± 0.12*	8
Cimetidine	1 μM	0.63 ± 0.17	0.29 ± 0.08	8
Thioperamide	1 μM	0.38 ± 0.12	0.27 ± 0.07	4
Cyproheptadine	30 nM	0.62 ± 0.21	0.07 ± 0.03*	8
Fexofenadine	1 μM	0.47 ± 0.05	0.08 ± 0.04*	8

*p < 0.05. Paired Student’s *t*-test.

## Discussion

Data obtained in this study has provided several novel findings to aid in our understanding of histamine’s influence on bladder contractility. There are four main findings. (1) Histamine causes an increase in baseline tension in both the U&LP and detrusor layers. (2) In U&LP, histamine not only increases the baseline tension but also influences the frequency and amplitude of spontaneous phasic contractions observed in the U&LP tissue. (3) Activation of muscarinic receptors, as suggested in previous literature^25^, is not involved in the contractile response to histamine. 4) H2 receptor activation stimulates relaxation of U&LP in response to histamine.

This study focused on the role of histamine receptors on the U&LP and detrusor, two distinct layers of the bladder. Previous research has identified increased mast cells within the lamina propria layers of those suffering from overactive bladder^15^. It is possible that degranulates from these infiltrating mast cells could be causing the observed inflammation via the release of inflammatory mediators such as histamine and prostaglandins^27^. In addition to mast cells, there are other immune cells of the body that are also capable of releasing histamine, such as basophils and neutrophils at sites of inflammation. Furthermore, biopsies of patients suffering from OAB have demonstrated clear evidence of chronic inflammation^17,28^ in histological samples. Previously, functional studies have shown that histamine is capable of causing contractions in human detrusor strips^29^, guinea pig bladder^21–23^ and rabbit detrusor^24^. However, our study is the first to identify and compare the responses to histamine in two distinct layers of the bladder that differ in structure and function: U&LP and detrusor. U&LP is comprised of several layers of epithelial cells (urothelium) that line the lumen of the bladder and the underlying connective tissue layer (lamina propria), whereas detrusor is made of smooth muscle cells.

It was determined that in the presence of the H1 antagonist pyrilamine, increases in baseline tension in response to histamine were significantly inhibited in both U&LP and detrusor preparations. This is consistent with past studies showing pyrilamine’s effectiveness in inhibiting contractions to histamine in rabbit detrusor tissue^30^ and cultured human detrusor cells^20^. It is known that U&LP is capable of developing spontaneous phasic contractions in absence of any stimulation^1^. Significant increases in these contractions were observed when tissues were treated with a single dose of histamine in the absence of any antagonists. However, when H1 receptor was blocked, the frequency of spontaneous phasic contractions did not increase. That indicates that H1 receptor not only is responsible for mediating the changes in baseline tension, but also in frequency of spontaneous phasic contractions in response to histamine. To further isolate the role of H1 in mediating contractions, combinations of antagonists were used. Whenever an H1 antagonist was present in the antagonist combination, increases in baseline tension in response to histamine were significantly inhibited. These findings ascertain the involvement of the H1 receptor in mediating contractions in the presence of histamine and further the conclusions from previous studies that present the involvement of H1 receptor in the mediation of bladder contractile responses^21,22^.

The role of the H2 receptor was established through agonist and antagonist studies. When tissues were treated with amthamine, which is a potent and highly selective H2 agonist, a relaxation of baseline tension was observed in U&LP samples, but not in detrusor preparations. The addition of an H2 antagonist, cimetidine, caused increases in baseline tension in U&LP, however had no effect on the frequency of spontaneous phasic contractions. The lack of responses to either the H2 antagonist cimetidine or agonist amthamine in detrusor preparation indicates that the H2 receptor subtype, even though present in the tissue, is not involved in detrusor contractions.

When the combined H3 and H4 receptor antagonist thioperamide was added to the U&LP, increases in baseline tension in response to histamine were observed. This increase in baseline tension most likely occurred due to the antagonist having an affinity for the other receptor subtypes present in the tissue. This was supported by experiments involving several antagonists. When U&LP was treated with all four receptor antagonists, the same small increase in baseline tension was observed, indicating that H3 and H4 receptors had no role in mediating the increase in tension. Previous literature suggests that increases in baseline tension to histamine, may be due a subsequent release of acetylcholine, acting on muscarinic receptors^25^ rather than an influence of histamine itself. However, the lack of any inhibition from atropine to the histamine response or receptor antagonism in this study suggests no involvement of muscarinic receptors. Any small increases in baseline tension observed in the presence of the H1 antagonist pyrilamine, are likely due to incomplete antagonism, as the concentrations were relatively small.

There were several differences between U&LP and detrusor tissue in response to histamine. It was determined that H1 and H2 receptors were only functional in U&LP tissue. Even though western blot analysis has shown all four histamine receptor subtypes present in the detrusor^20^, solely H1 was functional, and contractions were mediated by H1 receptors in both tissues. As epithelial cells of the urothelium have no contractile properties, the cells most likely involved in the contraction observed in organ bath experiments from U&LP tissue strips are the myofibroblasts or muscularis mucosa cells found in the lamina propria. Given the size and distribution of these cells within the connective tissue, they stimulate a strong contraction.

## Conclusions

Histamine influences the contractile activity of both U&LP and detrusor of the porcine urinary bladder. It was determined that stimulation of the H1 receptor results in contractions in both urothelium with lamina propria and detrusor tissue samples. Activation of the H2 receptor inhibits the H1-mediated contractions in the urothelium with lamina propria but not the detrusor, with H3 and H4 receptors having no functional role in bladder contractility. This study presents the H1 and H2 receptors as potential targets for future treatments for overactive bladder and other bladder contractile disorders.
